# Rabies Virus Antibodies from Oral Vaccination as a Correlate of Protection against Lethal Infection in Wildlife

**DOI:** 10.3390/tropicalmed2030031

**Published:** 2017-07-21

**Authors:** Susan M. Moore, Amy Gilbert, Ad Vos, Conrad M. Freuling, Christine Ellis, Jeannette Kliemt, Thomas Müller

**Affiliations:** 1 Kansas State University, Veterinary Diagnostic Laboratory, Rabies Laboratory, Manhattan, KS 66502, USA; 2 National Wildlife Research Center, US Department of Agriculture, Animal and Plant Health Inspection Service, Wildlife Services, Fort Collins, CO 80521, USA; Amy.T.Gilbert@aphis.usda.gov (A.G.); Christine.K.Ellis@aphis.usda.gov (C.E.); 3 IDT Biologika GmbH, 06861 Dessau-Rosslau, Germany; Ad.Vos@idt-biologika.de; 4 Friedrich-Loeffler-Institut, Federal Research Institute for Animal Health, Institute of Molecular Virology and Cell Biology, 17493 Greifswald-Insel Riems, Germany; Conrad.Freuling@fli.de (C.M.F.); Jeannette.Kliemt@fli.de (J.K.); Thomas.Mueller@fli.de (T.M.)

**Keywords:** rabies, wildlife, vaccination, antibodies, serology

## Abstract

Both cell-mediated and humoral immune effectors are important in combating rabies infection, although the humoral response receives greater attention regarding rabies prevention. The principle of preventive vaccination has been adopted for strategies of oral rabies vaccination (ORV) of wildlife reservoir populations for decades to control circulation of rabies virus in free-ranging hosts. There remains much debate about the levels of rabies antibodies (and the assays to measure them) that confer resistance to rabies virus. In this paper, data from published literature and our own unpublished animal studies on the induction of rabies binding and neutralizing antibodies following oral immunization of animals with live attenuated or recombinant rabies vaccines, are examined as correlates of protection against lethal rabies infection in captive challenge settings. Analysis of our studies suggests that, though serum neutralization test results are expected to reflect in vivo protection, the blocking enzyme linked immunosorbent assay (ELISA) result at Day 28 was a better predictor of survival. ELISA kits may have an advantage of greater precision and ability to compare results among different studies and laboratories based on the inherent standardization of the kit format. This paper examines current knowledge and study findings to guide meaningful interpretation of serology results in oral baiting monitoring.

## 1. Introduction

Rabies is caused by infection with lyssaviruses, which are a group of single-stranded negative-sense RNA viruses in the family Rhabdoviridae. Lyssaviruses principally infect the nervous system (NS) of mammals, and this infection is nearly 100% fatal in both reservoir and incidental hosts after a prolonged incubation period of 1–3 months [1]. It is well recognized that both cell-mediated and humoral immune effectors are important in combating rabies infection [2], although the humoral response receives greater attention with regard to prevention of rabies. Rabies virus neutralizing antibodies comprise an important part of the humoral response and are able to block virus entry into cells, thus preventing or limiting infection, and entry of the virus into the NS. While the administration of post-exposure prophylaxis (PEP) is restricted to humans, preventive prophylaxis is recommended or required for domestic animal hosts in many parts of the world, and the principle of preventive vaccination has been adopted for strategies of oral rabies vaccination (ORV) of wildlife reservoir populations for decades to control circulation of rabies virus in free-ranging hosts [3,4]. Efficacy of the biologic in protecting against a lethal rabies infection in a significant proportion of vaccinated animals (e.g., >86%) must be demonstrated in a captive setting prior to product licensure [5]. There remains much debate about the levels of rabies antibodies that confer resistance to rabies virus infection and, although antibody levels are one key index of resistance to challenge in animal models [6,7], no single cutoff level of rabies antibody is recognized as being invariably protective [8]. This is due to repeated observations that small fractions of animals presenting detectable levels of antibody prior to challenge can still succumb to rabies infection, and conversely some seronegative animals survive challenge [8,9].

The immunogenicity of the vaccine, measured by induction of rabies binding and/or neutralizing antibodies, generally correlates well with survival to lethal challenge, although there may also be significant yet unmeasured cell-mediated responses involved during infection and replication of the live vaccine virus in the oral mucosa and tonsils. An inverse relationship between the strength of the humoral versus cell-mediated immune responses to inactivated rabies vaccination in humans was documented in one study [10], and it is unclear whether animals demonstrate a similar inverse relationship following infection with live oral rabies vaccines. Along with other host factors, this in part also contributes to the inability of rabies antibodies alone to serve as a perfect correlate of protection against lethal infection. Studies have demonstrated the level of immune response to vaccination is related to an individual’s polygenetic major histocompatibility complex (MHC) phenotype [11,12,13]. Such genetic variation in peptide binding regions may also play a role in the induction of rabies virus antibodies [14]. Depending on the length of the experiment, rabies antibodies can be measured at several time points pre- and post-challenge, and are important for a greater understanding of antibody kinetics post-vaccination and anamnestic response induced following challenge. Despite this, two time periods appear to yield the critical information needed for predicting the survival of most vaccinated animals in efficacy studies: the initial induction or Days 15–30 post-vaccination antibody level, and the level of antibody present immediately prior to rabies virus challenge. Studies need only establish that the animals were naïve prior to vaccination, as ultimately the survival data alone following challenge determine vaccine efficacy. The gold standard in measurement of rabies antibodies has typically involved serum neutralization tests (SNT) to detect rabies virus neutralizing antibodies (rVNA) (i.e., the rapid fluorescent focus inhibition test (RFFIT) or fluorescent antibody virus neutralization test (FAVN)), although enzyme-linked immunosorbent assays (ELISAs) are now also recognized as acceptable tests to detect binding rabies antibodies. Few studies measure both by SNT and ELISA methods but, where they have, a strong but not strict correlation in levels is observed between these two different antibody detection methods [15,16,17,18].

Part of ORV post-campaign monitoring is assessing the vaccination coverage by demonstrating an immune response in the target species (population level antibody prevalence); animals are sampled during a pre-determined interval after bait distribution. The application of rabies serology for monitoring the immune response through sampling demands an understanding of the relationship of antibody measured and survival upon challenge. To date, no study has examined whether this sample measured by the prescribed assays with a set cutoff value is actually suitable in terms of predicting protective immunity in wildlife populations (population level immunity). Similarly, reliability of the prescribed assays has not been comprehensively established. The objective of this paper is to quantitatively examine the induction of rabies binding and neutralizing antibodies following oral immunization of animals with live attenuated or recombinant rabies vaccines, as correlates of protection against lethal rabies infection in captive challenge settings by reviewing both published literature and our own unpublished animal studies. Using these data, we challenge the notion of a single antibody cutoff level being used to indicate complete protection against lethal rabies challenge across animal species.

## 2. Materials and Methods

### 2.1. Literature Review

Published peer-reviewed journal articles concerning oral rabies vaccination in wildlife that included rabies antibody measurement after oral vaccination and challenge were reviewed in an attempt to identify a protective level per rabies antibody. These were obtained through a search on PubMed with the terms “rabies oral vaccination”, “rabies challenge studies in wildlife”. Of the 42 articles initially identified by description in the abstracts, 6 were removed for lack of serological data leaving 35 articles [19,20,21,22,23,24,25,26,27,28,29,30,31,32,33,34,35,36,37,38,39,40,41,42,43,44,45,46,47,48,49,50,51,52,53]. Six of the articles included studies on more than one species. The number of articles by species included fox (17), skunk (9), raccoon, (7), dog (6), and one each for mongoose and raccoon dog. The articles were reviewed for information about vaccination construct and challenge virus doses and strains, serological assay type, and sampling time periods, as well as correlation of antibody measurement with survival/death outcomes and are summarized in Table 1. In particular, identification of discrepancies and sources of potential errors as well as studies that could be comparable due to consistent study designs were targeted.

### 2.2. Animal Studies

Empirical data from 28 different animal studies were analyzed (Table 2). All vaccinated animals received vaccine constructs by oral route; either by direct oral instillation or by offering a bait containing a vaccine-loaded blister. Hence, the animal species included in Table 2 are all considered target species for oral vaccination.

### 2.3. Animals

Animals described in Table 2 were obtained from commercial sources, except for the small Indian mongoose (*Herpestes auropunctatus*). Mongooses were wild-caught on a rabies-free island off the coast of Croatia. All studies were carried out according to prevailing guidelines. All experimental protocols had been reviewed and approved by the German and American Ethics Committees. All studies at IDT were approved by “Landesverwaltungsamt Sachsen—Anhalt, Referat Verbraucherschutz, Veterinär-angelegenheiten”. All studies at Friedrich-Loeffler-Institut (FLI) were approved by “Landesamt für Landwirtschaft, Lebensmittelsicherheit und Fischerei Mecklenburg-Vorpommern”. Studies at NWRC were approved by NWRC institutional animal care and use committee (IACUC), see Table 2 for approval information (approval board, number, date).

### 2.4. Vaccine

Five different vaccine constructs were used in the empirical studies. The four rabies virus constructs were modified by site-directed mutagenesis using reverse genetics. The live recombinant virus construct expresses the rabies virus glycoprotein SPBN GAS [54], SPBN GASGAS [55,56], SAD dIND [57], ORA-DPC [58,59], and HAdVRG1.3 [60,61].

### 2.5. Serum Samples

Individual serum samples were used from experimental efficacy and challenge studies involving animals that were captive raised and never had prior vaccination against rabies, or that had been collected from a rabies-free area. In efficacy studies, depending on the experimental design, serum samples were taken at baseline and different time points post-vaccination prior to and post-challenge. For the purpose of this study, only serum samples collected at baseline (Day 0) and post-vaccination on Days 28–30 (referred to later as Day 28) and the day of or immediately prior to challenge virus administration were considered in the analysis. In challenge virus titration studies, blood samples were only taken at baseline prior to infection (Day 0). All sera were stored at −20 °C and heat inactivated after thawing for 30 min at 56 °C prior to testing. The samples were tested using a RFFIT (SNT) and ELISA assays. Sometimes no absolute value could be determined for the level of rVNA. In this case, and for the purpose of quantitative analyses, the upper limit was halved. For example, values less than 0.5 and 0.13 International Unit per milliliter (IU/mL) were converted to 0.25 and 0.07 IU/mL, respectively.

### 2.6. Serology

At the FLI, rVNA for each sample was determined by using a modification of the RFFIT (Smith et al., 1973) essentially as described [62] using the standard rabies (RABV) challenge strain, challenge virus standard (CVS-11), as test virus. For titration, a two-fold lg2 dilution series of each serum sample (1:10 to 1:5120) in Dulbecco minimum essential medium (DMEM) was prepared. Subsequently, 50 µL of the serum dilution was mixed with 50 µL of a constant dilution of the test virus (adjusted to induce about 40 infected cells in a well of a Terasaki plate). After incubation for 90 min at 37 °C, 0.1 mL of a 10^6^/mL concentration of baby hamster kidney (BHK)-21 C 13 cells in DMEM containing 10% (wt/v) fetal calf serum was added. Subsequently, 10 µL of the serum-virus-cell suspension was pipetted into wells of a Terasaki plate (Greiner, Germany) and incubated for 24 h in a controlled humidity, CO_2_ incubator at 37 °C. After fixation with acetone (80%) for 30 min at 4 °C, cells were stained with a fluorescein isothiocyanate (FITC) anti-rabies conjugate (Fujirebio Diagnostics, Malvern, PA, USA) for 30–60 min and examined with an inverse immunofluorescence microscope. In each test run, the World Health Organization (WHO) international standard immunoglobulin (2nd human rabies immunoglobulin preparation, National Institute for Biological Standards and Control, Potters Bar, UK) adjusted to 0.5 IU/mL, and a naïve serum, served as a positive and negative control, respectively. The rVNA titer was defined as the dilution of the test serum showing a 50% reduction in the number of infected cells per microscopic field (50% neutralizing dose, ND50) compared to the virus control. The exact titer was calculated using inverse interpolation as described [63] and subsequently converted into concentrations expressed in international units (IU/mL) using the calibrated positive control. As a national and OIE reference laboratory and WHO Collaborating Center for rabies the FLI is certified by the German accreditation body (DAkks) for human and animal rVNA testing and recognized by the German Ministry of Agriculture for performance of rVNA measurement of animal sera for pet travel purposes. The RFFIT assay described here has been internally validated per OIE guidelines for the purpose of rVNA measurement and per governmental approval, as it had been officially used for monitoring of oral vaccination campaigns of foxes in Germany for many years. Sensitivity and specificity was 95% and 98%, respectively.

At Kansas State University, rVNAs were measured in serum samples following the RFFIT method [64] as published in the WHO and the World Organisation for Animal Health (OIE) manuals [5,65]. Briefly, 100 µL of each serum sample, in duplicate, was diluted in serial five-fold dilutions in 10% fetal bovine serum supplemented Eagles minimum essential media (EMEM) and loaded into 8-well lab-tek chamber slides after which 100 µL of the challenge virus, at a concentration of 50 tissue culture infective dose 50% (TCID_50_), was added. Slides were incubated at 37 °C for 90 min after which 200 µL of a suspension of 5 × 10^5^ BHK-21 C 13 cells in 10% FBS supplemented EMEM was added to each well. Slides were placed in a 2–5% CO_2_ incubator at 37 °C for 24 h. After incubation, the slides were washed and fixed in 80% cold acetone, dried and stained with FITC conjugated anti-rabies antibody (Chemicon, Temecula, CA, USA). Twenty fields/well were examined under 100× magnification using a fluorescence microscope for the presence of rabies virus and rVNA titers were calculated using the Reed and Muench method [66]. In each test run, an international standard rabies immunoglobulin (SRIG) (WHO 1st human rabies immunoglobulin preparation Lot R-3, FDA/CBER, Rockville, MD, USA or WHO 2nd human rabies immunoglobulin preparation, National Institute for Biological Standards and Control, Potters Bar, UK) adjusted to 2.0 IU/mL, internal rVNA standards, and naïve serum served as controls. The SRIG was also used as a calibrator to calculate the test sample IU/mL value by the flowing formula: SRIG titer/Sample titer × SRIG potency in IU/mL. The RFFIT assay has been internally validated for rVNA measurement at Kansas State University (KSU) to international standards for human samples [67] and per OIE guidelines and United States Department of Agriculture (USDA) recognized for the purpose of pet travel and wildlife serosurveys. Sensitivity and specificity was 98% and 98%, respectively.

Sera were examined in a commercial blocking ELISA (BioPro Rabies ELISA, BioPro, Prague, Czech Republic) [68] for the presence of rabies-specific binding antibodies, following instruction of the manufacturer essentially as described [69]. Briefly, 100 µL of a 1:2 dilution of each serum was pipetted into wells of full RABV antigen coated microplate. After overnight incubation at 2–8 °C, in two consecutively-following steps 100 µL of a biotinylated anti-rabies antibody and 100 µL horseradish peroxidase (HRP)-conjugated streptavidin was added to each wells and incubated at 37 °C for 30 min followed by four times rinsing to remove unbound conjugate. Color change was developed by adding 100 µL of 3,3′, 5,5-tetramethylbenzidine (TMB) chromogen solution to each well for 15–20 min at room temperature and the quantity of the analyte measured at 450 nm. According to the manufacturer, a 40% inhibition of the test serum compared to the negative controls was considered a cutoff for positivity and a 70% inhibition considered equal to 0.5 IU/mL. The performance characteristics were evaluated by the manufacturer and stated in the kit insert. The BioPro ELISA kit has been internally validated at FLI for humans, foxes, raccoon dogs, mongooses and raccoons using defined experimental (vaccinated/unvaccinated) as well as field sera.

A subset of sera was examined with a commercial indirect ELISA (Bio-Rad Platelia Rabies Kit II Ref: 355 1180, Marnes-la-Coquette, France) using the Bio-Rad Evolis instrument per the manufacturer’s instructions. The kit contains strips of wells coated with rabies glycoprotein (G protein). The secondary (detection) system is an enzyme conjugated *Staphylococcus aureus* protein A/substrate color reaction. The results were reported in equivalent units (EU)/mL (anti-rabies glycoprotein level) calculated by comparison of the sample optical density reading against a standard curve of positive standards supplied in the kit. The kit control is calibrated against the 2nd WHO (World Health Organization) international rabies immunoglobulin reference serum. The performance characteristics were evaluated by the manufacturer and stated in the kit insert [70]. The BioRad ELISA kit has been internally validated for use with raccoon sera at KSU.

### 2.7. Statistical Analysis

Data were analyzed using statistical software GraphPad Prism version 7.00 for Windows, GraphPad Software, La Jolla, California, USA. Test agreement was calculated using the GraphPad QuickCalcs Web site: http://graphpad.com/quickcalcs/kappa2/.

## 3. Results

### 3.1. Literature Review

#### 3.1.1. Serological Methods and Study Design Summary

The vaccines studies covered a combination of live attenuated and/or genetically altered rabies viruses: SAD, SAG, and ERA; and live recombinant constructs incorporating only the rabies virus glycoprotein: VRG, HAdV5RG1.3, Ad5RG1, and CAV2-RVG, as well as mutants of these. Most are linked with the original SAD isolate in 1935. In general, the challenge virus strain used was derived from the species under study, with seven exceptions (Table 1). The challenge dose varied between 1 × 10^3^ and 1 × 10^8^ MICLD_50_. The site of challenge was mostly the masseter and temporal muscles, but also targeted were the biceps, gluteal, abductor digiti quinti, and cervical musculature. The interval between vaccine and challenge ranged from 28 to 2490 days.

In these studies (Table 1), the RFFIT was the most frequently employed assay for measurement of rabies antibody levels in response to vaccination, and the majority (but not all) referenced the original method in Smith, 1973. The remaining studies, besides three using a competitive ELISA (cELISA), used other serum neutralization assays: four used FAVN method as described in the OIE manual; five used a fluorescence inhibition microtest (FIMT) that essentially represents a modified RFFIT [71], three used other modified RFFIT methods, and three used undefined serum neutralization assays. Only 54% (19 of 35) of the papers reported a cutoff level of seroconversion or seropositivity. In ten of the 19 studies, 0.5 IU/mL was the level defined as rVNA-positive; these included studies using RFFIT (4), FAVN (4), and modified RFFIT (2) assays. Three of the studies using FIMT applied 0.13 IU/mL as the level of seropositivity. Three referred to evidence of neutralization at a 1:5 dilution of the serum, but also reported the result in IU/mL. The three studies using the cELISA assay defined seroconversion as ≥25%, 26% and 28% inhibition, and one further defined it as three consecutive weeks of measurements above the cutoff, and measured antibody response by cELISA in weekly samples, but the pre-challenge samples by SNT.

#### 3.1.2. Serological Results Summary

Due to the heterogeneity of experimental design and serological methods used, after review of the published studies listed in Table 1, the relationship of vaccinated status to serology and survival following challenge could only allow a general conclusion: the majority of the subjects in the studies that developed detectable rabies antibodies post-vaccination were also more likely to survive challenge. Those individual subjects with the highest titers in each separate study had the best likelihood of survival regardless of the range in titers produced in the vaccinated subjects, demonstrating that, though the assays used in these various studies had not been formally correlated, and indeed could vary in cutoff level and range, the results were generally consistent in identifying the likely survivors in a group by the level of antibody measured. Vaccinated status did not guarantee survival, and neither did demonstration of circulating rabies antibodies post-vaccination.

Thirty-five studies reported rabies antibody level in individual values, either IU/mL, titer (for SNT assays), or percent inhibition (for cELISA assays). In regards to correlation of serology results to survival from challenge, in 18 of the 39 studies (46%) the relationship was absolute: all subjects with detectable rabies antibodies survived challenge and all with no detectable antibody succumbed. For 66% (12/18) of those, the effective cutoff level was 0.5 IU/mL. Animals with undetectable or below cutoff rVNA levels survived challenge in 14 of the 39 studies (36%). However, in 6 of the 39 studies the opposite trend occurred where rVNA “positive” animals succumbed (e.g., levels of 0.65 IU/mL, 0.57 IU/mL, >0.5 IU/mL, 1.07 IU/mL, 1.3 IU/mL, and >1:5 titer); in many of these events, the animal that succumbed had the lowest rVNA level among the group of vaccinated subjects in the experiment. Only in one of the 39 studies was the relationship between serological response and survival weak; subjects that succumbed had rVNA levels at day of challenge between 0.06 and 2.2 IU/mL and, for the ones that survived, the range was 0.04 to 12.2 IU/mL. In this study, the subjects were considered seroconverted if they had detectable rabies antibodies for more than three consecutive weeks; there was no correlation of seroconversion to survival, with the range of consecutive weeks for both the survivors and died encompassing 1–15 weeks.

The ability of the rabies serology results to predict percent survival is partially predicated on the day of sampling and the day of challenge. In the 26 studies where both Days 14–30 and day of challenge (within seven days) rabies antibody results were given, Days 14–30 antibody levels were higher or essentially the same as at day of challenge in 69% of the studies; the levels higher at day of challenge in 31% of the studies. For the remaining studies where the results were higher at the earlier time-point, the day of challenge occurred later than 90 days post-vaccination, thus in extended sampling it appears the determination of early seroconversion was a better predictor of survival than the level at day of challenge.

### 3.2. Animal Studies

#### 3.2.1. Correlation between Blocking ELISA and RFFIT Values and Test Agreement

**Figure 1:**
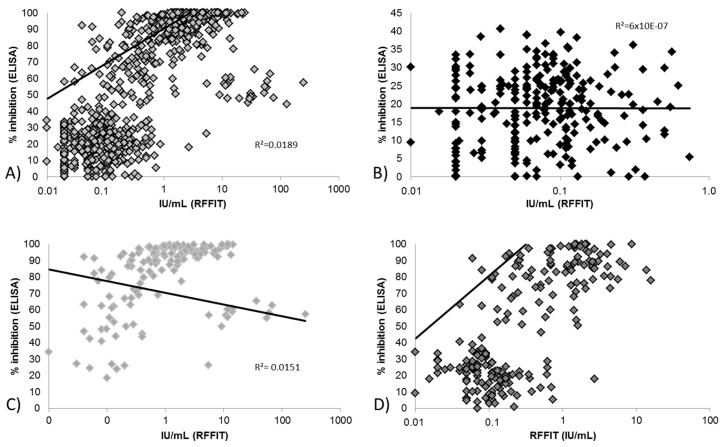
Correlation between the rapid fluorescent focus inhibition test (RFFIT) (IU/mL) and blocking enzyme linked immunosorbent assay (ELISA) (% inhibition) results of: (**A**) pooled time points and species (*n* = 725); (**B**) pooled species baseline samples (*n* = 255); (**C**) pooled species day of challenge samples (*n* = 178); and (**D**) pooled time points fox samples (*n* = 212). Blocking ELISA values of <0% and >100% were set at 0% and 100%, respectively.

For pooled data independent of species (Figure 1) there was a poor correlation (R² = 0.0189) between ELISA and RFFIT values, although a significant positive trend is observed (*p* = 0.0002; 95% CI of slope 0.1792 to 0.5760, Figure 1A). The correlation remains poor even if different subgroups, i.e., baseline versus day of challenge sera, are considered. For baseline samples, there was no detectable trend (Figure 1B–D). Only for the red fox (*n* = 212), was a significant positive trend detected (*p* <0.0001; 95% CI of slope 5.569 to 9.850, Figure 1D).

**Figure 2:**
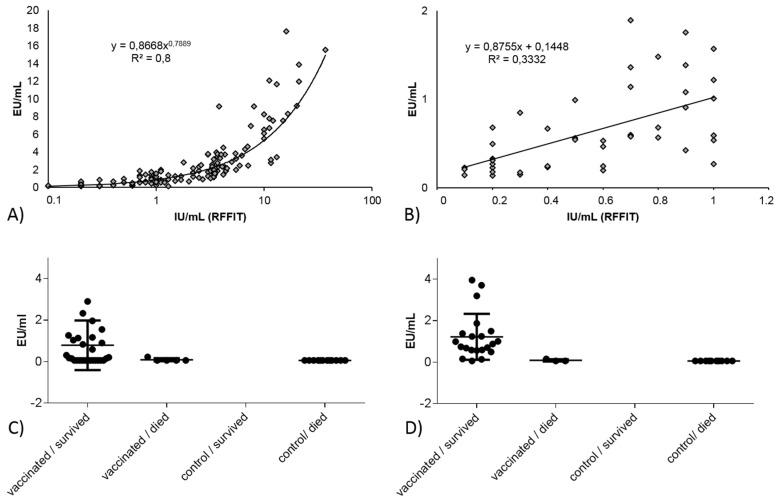
The correlation between RFFIT and indirect ELISA (Bio-Rad) for study 8 (Table 2): (**A**) the complete data set of absolute values, one outlier (indirect ELISA—48.78) was removed; (**B**) same data set but only for values with a RFFIT-result of <1.0 IU/mL; (**C**) indirect ELISA results at day of challenge for the vaccinated and control animals per outcome of challenge; and (**D**) indirect ELISA results on Day 28 post-vaccination for the vaccinated and control animals per outcome of the challenge infection. EU/mL is Equivalent Unit per milliliter.

In studies 8 and 9 (Table 2), an indirect ELISA (Bio-Rad) was used. A better correlation between the RFFIT and the indirect ELISA values was demonstrated overall, although not linear (Figure 2A). However, when the correlation was determined for RFFIT values ≤1.0 IU/mL, the correlation of this subset is relatively poor (Figure 2B). While it appears that the Bio-Rad ELISA is quite suitable in identifying true negatives, the test had difficulties in identifying vaccinated animals, particularly at Day 28 (Figure 2D). In total, 12 of 29 (41.4%) vaccinated raccoons in this study tested “negative” (≤0.125) in the Bio-Rad ELISA on day of challenge but subsequently survived the challenge infection (Figure 2C).

#### 3.2.2. Limit of Detection for Blocking ELISA and RFFIT

**Figure 3:**
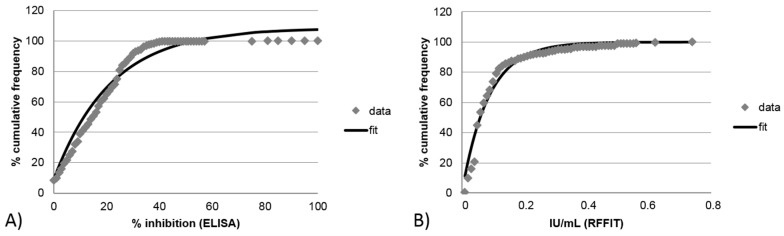
Cumulative frequency of rabies antibody test values in samples from unvaccinated animals (i.e., baseline samples of vaccinated animals and control unvaccinated animals) for the: blocking ELISA (**A**); and the RFFIT (**B**). For the latter, only absolute values were used and relative values of <0.5 or <0.1 IU/mL were omitted.

For both assays, the lower limit of detection was assessed by analyzing the immune response measured in naïve (unvaccinated) animals. For the blocking ELISA the equation of best fit was y = 8.44 + (1−e^−0.0476X^), resulting in 95% of all samples from unvaccinated animals showing an inhibition of <43% (Figure 3A). When the same analysis was done for RFFIT results using the same sample set, the equation of best fit was y = 100(1−e^−11.79X^). In this case, 95% and 70% of all samples from unvaccinated animals had rVNA-levels of <0.25 and <0.10 IU/mL, respectively. Hence, the lower limit of detection for RFFIT was <0.25 IU/mL (Figure 3B).

#### 3.2.3. Correlation and Agreement of Test Results Obtained in Blocking ELISA and RFFIT with Survival/Death

Based on the currently used thresholds of seropositivity (blocking ELISA: cutoff >40% inhibition; RFFIT >0.5 IU/mL), the agreement between the assays was high (85.66%) resulting in a Kappa value of 0.71 (95%CI: 0.66 to 0.76). When the RFFIT cutoff was set as >0.25 IU/mL, the agreement slightly increased to 0.75 (95% CI: 0.70 to 0.80). In both cases, the strength of agreement was considered to be “good” (Table 3).

**Figure 4:**
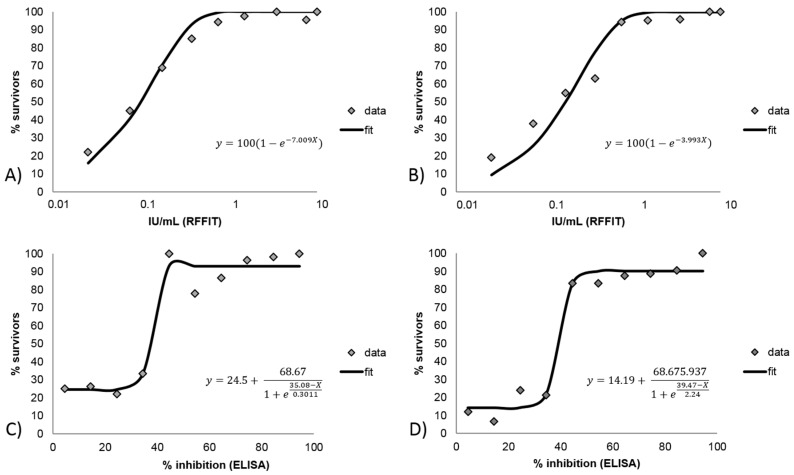
Relation between survivorship and immune response at: Day 28 post-vaccination (**A**,**C**); and the day of challenge (**B**,**D**). For RFFIT (**A**,**B**) the Michaelis Menten model is shown, while for blocking ELISA (**C**,**D**) the Boltzmann sigmoidal model is shown.

When the immune response measured at Day 28 post vaccination, day of challenge, and the outcome of the challenge were analyzed using RFFIT values, a Michaelis Menten model demonstrated the best fit (Figure 4A,B). This model predicts a survivorship of 95% and 99% of the animals with rVNA levels of ≥0.43 and 0.65 IU/mL at Day 28 post vaccination, irrespective of day of infection, respectively. In contrast, for the same time points and outcomes but using blocking ELISA values, a Boltzmann sigmoidal model demonstrated the best fit (Figure 3C,D). Here, survivorship of 93% and 57% are predicted when animals have inhibition values of ≥40% at Day 28 and at the day before challenge, respectively.

When comparing different thresholds of seropositivity for both assays, the relative chance of a seropositive animal to survive a challenge compared to a negative animal was highest for the blocking ELISA with the currently defined cutoff of 40% inhibition. In addition, the sensitivity was highest for this cutoff setting, while still demonstrating sufficient specificity (Table 3). While for all cutoff settings the positive predictive value was higher than 95%, the negative predictive value was again highest for the blocking ELISA with the cutoff of 40% inhibition. Although the agreement between test result and outcome of infection varied across species, the best agreement (i.e., “good”) was achieved for the 40% inhibition test setting.

**Figure 5:**
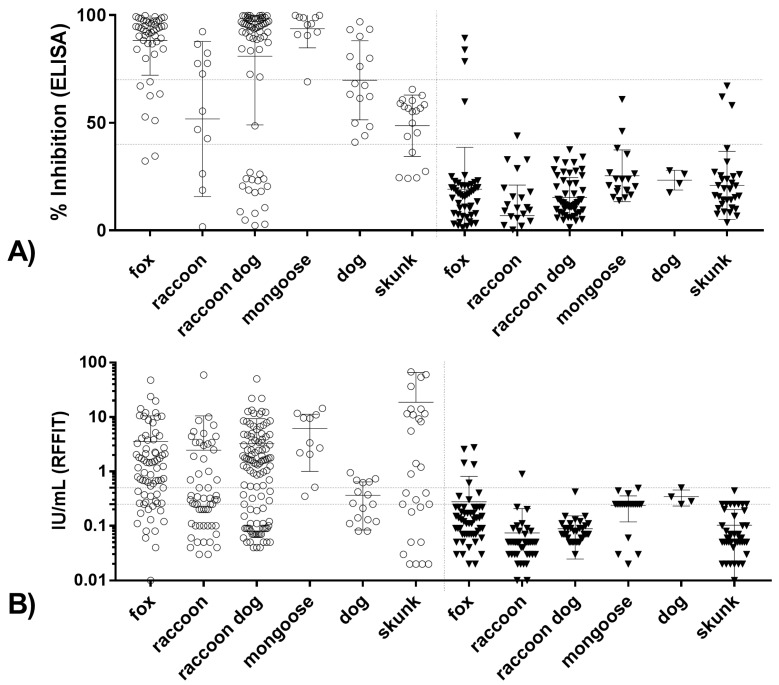
Scatter dot plot for: blocking ELISA (**A**); and RFFIT (**B**), for the individual species. Values are presented for survived (open circles) as well as for animals that succumbed to the infection (black triangle). Mean and standard deviation are indicated. Horizontal dotted lines indicate the thresholds used for blocking ELISA (40% and 70% inhibition) and for RFFIT (0.5 and 0.25 IU/mL).

**Figure 6:**
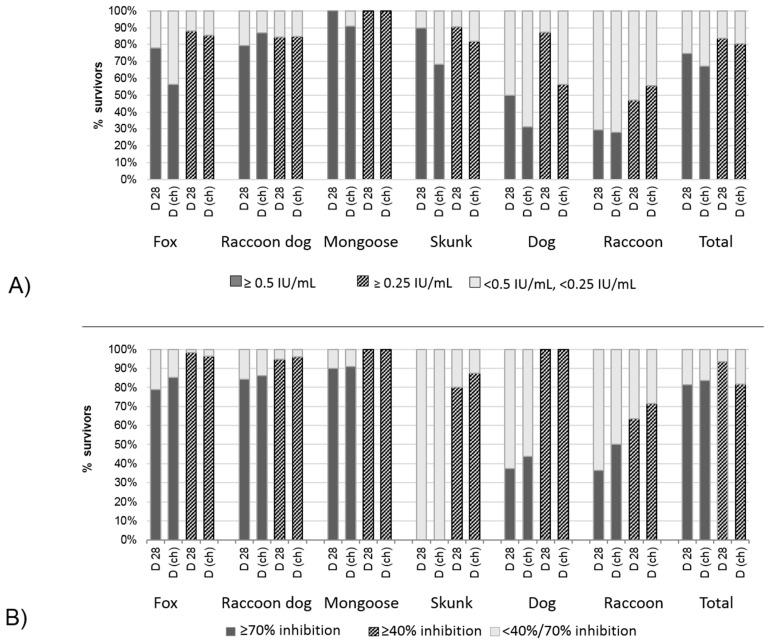
(**A**) Percentage of RFFIT positives and negatives per species among survivors, at different time points and using different cutoff values; and (**B**) percentage of blocking ELISA positives (≥40% inhibition) and negatives per species at Day 28 post-vaccination (D 28) and before challenge(D (ch)).

The good agreement with a cutoff of 40% inhibition for the blocking ELISA was also evident when species were analyzed separately, where except for raccoon dogs, very few animals with values <40% survived a challenge infection (Figure 5 and Figure 6). In contrast, low rVNAs (0.05 to 0.25 IU/mL) were obtained in both survivors and animals that succumbed to rabies challenge. Some species-level differences were evident, with most survivors among mongooses having RFFIT titers ≥0.5 IU/mL, while this proportion is lowest for dogs and raccoons. Except for raccoon dogs, where the proportion of RFFIT positives among survivors remains the same, a lower cutoff value of 0.25 IU/mL increases the proportion. The same applies to the day of testing, where samples taken 28 days post-vaccination have a greater proportion of RFFIT positive results than samples taken at the day of challenge (Figure 6A). This effect is not evident for the blocking ELISA, which generally provides a better match between survivorship and a positive result. Again, similar to the RFFIT, raccoons had the highest proportion of blocking ELISA negatives among survivors (Figure 6B).

## 4. Discussion

Challenge studies demonstrating oral rabies vaccine efficacy in animals follow the same study design standards as parenterally-administered live or inactivated rabies vaccines. However, for oral vaccines, two efficacy studies are recommended by OIE guidelines [5], one involving direct instillation of the vaccine into the oral cavity, and the other involving bait delivery of the vaccine. However, U.S. and European specific guidelines appear to only require demonstration of bait efficacy for licensing [72,73]. The duration of vaccine immunity may vary according to product label intent and applicable guidelines. The minimum recommended duration standard for wildlife oral rabies vaccines is 180 days in the European Union [5] and 365 days in the U.S. [72]. Other differences include blood sampling at different time points and serological definition of population-level rabies immunity. For example, the US standards require several serum antibody measurements post-vaccination and prior to challenge [72], whereas the European Union does not [73]. Additionally, the proportion of vaccinated animals surviving challenge must be 87% or greater in the U.S. and with 80% or greater mortality of unvaccinated controls, but the European Union requires 92% or greater survival among vaccinates and 90% or greater mortality of unvaccinated controls [72,73]. These differences in acceptance standards pose difficulty for industry and end-users in defining adequate efficacy monitoring levels.

Tools to assess the effectiveness of ORV baiting programs to control rabies in an area are currently limited to measurement of rabies antibodies produced in response to vaccination, or case reductions or elimination of rabies cases following successive campaigns as the most definitive proof of baiting program effectiveness. However, particularly in rabies-free vaccination zones implemented to stop further spread, there is no other tool to verify effectiveness than to test population-based immunity. To this end, population-level antibody prevalence still is an imperfect means for assessing ORV baiting efforts, as antibodies are not the only informative measure of immunity against rabies infection. Clearly, the cellular as well as humoral immunity has been shown to play an important role in preventing disease, as well as innate immunity. However, the key to control rabies in susceptible wildlife is pre-exposure vaccination. This is due to unique viral mechanisms of the rabies virus. Pathogenic wild type rabies virus likely limits replication in nerves to lower the expression of glycoprotein, and downregulates receptors and signaling for infected nerve apoptosis by the immune system [74,75,76]. The virus also appears to downregulate the inflammatory response and enhance destruction of infiltrating lymphocytes to actively suppress the immune system [74]. Prior to accessing the NS, humoral and cell-mediated immune effector cells may have an opportunity to detect and clear the virus, which may permit host survival. In this regard, the type 1 helper T cell pathway of response, signaled by increased production of interferon gamma (IFN-γ) and inflammation, is critical to activation of cytotoxic T cells for clearance of the peripheral virus infection. However, nerve cells do not express class I MHC for presentation to activated T cells, and once rabies virus enters the immune-privileged central nervous system (CNS), the immunosubversive and evasive strategies of wild type RABV make it nearly impossible for the host to effectively combat the NS infection [2,74]. Vaccination prior to virus challenge induces immune effectors (B and T cells) for establishment of rabies-specific antibody producing plasma cells. Circulating rabies antibodies will then be present to neutralize virus at the time of exposure as well as memory cells, which are primed and ready to respond and expand the response on repeated activation [77]. This effectively eliminates immunosubversive and evasive mechanisms of the rabies virus.

Oral vaccination of wildlife has led to the elimination of rabies in the target species in large parts of Europe and North America [78]. Application of serological monitoring as means of evaluating vaccination efficacy demands good understanding of the relationship of measured antibody level and survival from challenge. Certainly there are host factors that affect individual response to vaccination (e.g., genetic variation in MHC molecules) in addition to variable bait uptake interactions, but in strictly considering the means of predicting successful immunization (protection), establishing the reliability of serological monitoring as a tool is critical. The review of previous studies in which serological testing was performed in rabies challenge studies presented several difficulties in deducing what rabies antibody level was associated with a strong probability of survival. Analysis of the serological results in the literature review indicated a general positive correlation between level of rabies antibody and survival, but also revealed several variations in measurements, and in some studies, no solid relationship between antibody level and survival was demonstrated [24]. In regard to the assays used, understanding the abilities and limitations of the assay as well as use of quality control measures aids in interpretation of results [79]. While it is true that each study evaluated in the literature review did not use the same vaccine, same strain and dose of challenge virus, or same day of challenge, it is also true that use of a consistent method to measure rabies antibody would have allowed more robust comparison of antibody protection elicited by vaccination across studies. There are critical components of serum neutralization assays that will directly affect the accuracy of the antibody measurements. Key among them are the virus dose, virus strain, and reference serum [80]. Use of a high dose of virus may not allow detection of low antibody levels and too low a dose may overestimate the level. Use of a challenge strain that is not closely related antigenically to the vaccine strain may underestimate the antibody level [81]. Additionally, if the reference serum used to calibrate the titer results is not an international standard or qualified against one, the resulting values may be skewed [8,82]. Therefore, at minimum, the specificity, sensitivity, and measureable range of the assay employed should be defined and identified when reporting results to allow discernment as to the comparability of the findings with other studies.

In the publications reviewed, the majority of serological measurements were performed with serum neutralization assays, which functionally should translate most clearly to protection. However, serum neutralization assays are less likely to be standardized when compared to ELISA assay kits, due to the many manual steps and component parts that can be independently sourced, thus less strictly controlled between laboratories. Therefore, while ELISA kits measure binding antibodies, not necessarily neutralizing antibodies, they have the advantage of ease of standardization and greater assurance of consistent results [8]. Several studies have demonstrated a good correlation between SNT and ELISA methods with human and animal sera at specific cutoffs [15,17,18,69,83,84]. In particular, a comparison of SNT and blocking ELISA in wild-caught raccoons and skunks from ORV areas demonstrated the utility and comparability of both assays at a cutoff of 0.5 IU/mL and 40% (or 25% as defined in some studies) respectively [83,85]. Overall, correlation between blocking ELISA and RFFIT values in this study for the all samples was poor, indicating that the two assays are measuring partially different antibody functions. This remains true even when the samples are analyzed in subgroups (survivors, died, Day 0, day of challenge, etc.) (Figure 1). Using the threshold for seropositivity (per BioPro, >40% inhibition; per RFFIT, the commonly used level of 0.5 IU/mL) good agreement between tests was obtained (Table 3). If a lower level was used for RFFIT seropositivity (0.25 IU/mL) the agreement was marginally improved. Correlation of BioRad ELISA and RFFIT results varied by day of draw with better correlation at day of challenge compared to Day 28, due to the fact that the ELISA only detects IgG antibodies, thus under-measures the total antibody response in the earlier time-point (Figure 2). Due to the simplicity, better precision, and repeatability of an ELISA kit compared to a complex, manual serum neutralization test, the functional test may not be the best test for the purpose of monitoring ORV campaigns. However, it is important to note ELISA techniques can vary in terms of ability to estimate protective (neutralizing) antibodies.

The key motivation for this study was to estimate a cutoff level associated with survival in animals vaccinated orally with live constructs. As can be surmised from the lack of correlation between seroconversion as assigned by cELISA results and challenge survival in one of the studies in the review [85], this is not an easy task. One of the complicating factors is species-associated matrix differences that can cause false positive results if the lower limit of detection has not been identified with a specific species serum, if there are interfering factors that prevent binding of competitive rabies antibodies in the cELISA or that non-specifically bind in the indirect ELISA [8,86]. The timing of blood draw for evaluation of antibody level appears to be critical in assessing protection conveyed by vaccination. Antibody development after vaccination has been well studied: a general pattern of detectable antibody level followed by a rise in neutralizing antibody, peaking between 14 and 30 days, followed by a slow decrease to a stable level; and if a booster dose is received a faster rise in level is expected followed by stabilization of a higher level [26,87]. While there are studies in dogs and cats that show a clear association between detectable rVNA before challenge and survival [6,7], review of available published studies in wildlife and our own empirical studies included here indicate that detection of rVNA at Days 28–30 is also predictive of survival, and usually more so the later the day of challenge was in relation to the day of vaccination. In a study where the challenge day was 83 months from vaccination, 60% of the survivors had detectable rVNA at challenge and 40% did not; of those 40%, all had detectable rVNA 66 months but only half of those had a level above 0.5 IU/mL at every sampling time point beyond the initial measurement at one month, indicating that measurable initial response was the better predictor of survival at challenge [31].

Determination of applicable cutoff level is important, as stated previously, for a specific assay given a specific purpose. Evaluation of background signal of negative samples provides information regarding a lower limit of detection. Ideally, negative samples will have levels significantly below the determined effective level to allow discernment between “positive” and “negative” samples. The same is true for determination of vaccination success and failure. Evaluation of Day 0 (unvaccinated subjects) samples (all species) revealed that 95% had a blocking ELISA result of ≤43% inhibition and a RFFIT result of ≤0.25 IU/mL. Species differences were noted, with raccoons having less of a variation in result range than other species (Figure 5), indicating that further evaluation of negative samples by species is necessary for assignment of cutoffs for lower limit of detection of an assay. Figure 4 illustrates that the blocking ELISA “negative” and “positive” results are more clearly delineated in scale compared to the RFFIT data, which shows a more gradual relationship between rVNA level and survival. For example, the statistical probability for survival increases dramatically between blocking ELISA values of 35% (30–40%) and 45% (40–50%) inhibitions, whereas the same increase in probability occurs between an order of magnitude difference (i.e., 0.06 and 0.5 IU/mL) for RFFIT values. For some species this may make assignment of a cutoff more difficult for RFFIT results than for the ELISA results (Table 1). This may indicate that the RFFIT is more sensitive at lower levels of antibody, causing poor delineation between “negative” and “positive” samples. Use of a specific challenge virus strain or increasing the challenge dose in the RFFIT procedure, possibly could correct this weakness. For RFFIT at >0.50 IU/mL, the probability of survival reaches 95% and at levels >2.0 IU/mL it becomes 100% using a best fit curve of the data. For the blocking ELISA, >80% probability of survival is obtained at >40% inhibition and at >70% inhibition over 90% probability of survival is attained. This analysis is remarkably similar whether the result used is from Day 28 or day of challenge, and our empirical results also demonstrated that Day 28 post-vaccination titer levels were a better predictor of survival than titers measured immediately prior to challenge. As expected, the higher the antibody level measured, whether by indirect ELISA, cELISA, blocking ELISA or RFFIT, the higher the probability of survival, to a robust point where the level is associated with 100% survival; beyond that point, higher titer levels do not translate into “better protection”.

Oral vaccination has proven to be an effective tool in rabies prevention and control. The target species varies per endemic region and vaccines are developed that are of highest efficacy in their targeted species. The specific G protein, copies presented, and other details of the vaccine construct in combination with the specific assay used for antibody measurement theoretically can affect the relationship of rabies antibody level and probability of survival. An analysis of the results by vaccine construct indicates constructs are not the reason for the effect seen in raccoons and skunks, and that the limited data for dogs do not allow for a robust conclusion (data not shown). It is not unusual therefore to see various correlations of serological results and survival among different species. Indeed, the results of this study demonstrate that a single cutoff level of seropositivity is not universally applicable. As mentioned previously, the unvaccinated raccoon samples had a lower mean percent inhibition by blocking ELISA and lower mean IU/mL by RFFIT compared to the other species (Figure 5A,B), while unvaccinated fox samples had a small number of samples with results above the cutoffs for both methods. For all the species, except fox, the 70% inhibition cutoff for blocking ELISA was robust; the lower threshold (40% inhibition) allowed misidentification of unvaccinated fox, raccoon, mongoose, and skunk samples. For RFFIT, the 0.25 IU/mL cutoff was robust for raccoons, raccoon dogs, and skunks, and the higher cutoff of 0.5 IU was better at identifying unvaccinated mongoose and dog samples (Figure 5A,B). The Bio-Rad ELISA results appeared to be better correlated to RFFIT results overall; however, for results below 1.0 IU/mL, the correlation is poor, indicating that it may not be as useful in situations where low antibody levels are expected. The Bio-Rad ELISA was quite suitable in identifying true negatives, while the test had difficulties in identifying vaccinated animals (Figure 2C,D). It is possible that increasing the sensitivity of this assay by adjustment of conjugate or antigen could improve performance for wildlife species.

These findings illustrate the importance of evaluation of the appropriate cutoff by species and by assay. Similarly, relationship of rabies antibody level and challenge outcome varied by species, assay used, and cutoff level. For RFFIT the best predictor of survival having a result of ≥0.25 IU/mL at Day 28 across species, although for raccoon dogs and raccoons the best predictors were ≥0.25 IU/mL at day of challenge (Figure 6A). For the blocking ELISA results, the 40% inhibition cutoff was an excellent predictor of survival for fox, raccoon dog, mongoose, and dog, with 90% or more subjects at that inhibition level surviving challenge. Across species, the 70% inhibition level was a less accurate predictor of survival (Figure 6B). Overall, the >40% inhibition blocking ELISA result at day of challenge determined survival most often, with the majority of results from fox and raccoon dog studies.

## 5. Conclusions

Acceptance standards for challenge studies determining an associated protective antibody level with challenge survival in the review of previous challenge studies in wildlife were not feasible (Table 1).

Assays must be evaluated for purpose (for example, to identify vaccinated animals, to identify vaccinated animals with protective immunity, and to identify free-ranging wild animals previously exposed to rabies infection) and generalizations should not be made to other purposes for which an assay was never evaluated. 

Specificity (based on the vaccine antigen and assay antigen relationship), sensitivity, accuracy (including linear range) and precision of different assays may vary among species. Standardization and quality control of reagents and procedures, whether kits or manual procedures are established, is absolutely essential. These criteria should be evaluated before assigning the assay as fit for purpose.

Timing of sampling for antibody response to oral vaccine bait uptake should be optimized to target the initial seroconversion peak period, because this point predicts (given the appropriate cutoff and assay for the target species) survival as well as or even better than the level measured just before challenge.

In our study, positive blocking ELISA results are a better predictor for survival than the various SNTs applied overall. If the ELISA kits used have sufficiently good lot to lot quality control, the results from one study can be directly compared to another separated by time and space, with high confidence. Monitoring of ORVs based on seroconversion rates of the target population should make use of blocking ELISA tests instead of SNTs, for both strength as a survival predictor and ease of standardization.

Whatever assay is selected, it should have a defined (by validation) relationship to protection and robustly meet the minimum requirements for the purpose.

## Figures and Tables

**Table 1:** Animal vaccine efficacy and challenge studies referenced for serology data as part of the literature review. MICLD_50_: Mouse intracerebral lethal dose 50%. See key for facility below.

Study	Animal Species	Number Vaccinated	Number Controls	Challenge After … Days	Challenge Dose (MICLD_50_ log)	Challenge Virus Strain	Facility Where Research Was Done	Reference
1	arctic fox	6	5	56	5.7	fox	5	30
2	arctic fox	8	4	112	5.7	canine/fox	5	27
3	arctic fox	10	4	56	3.9	fox	5	28
4	arctic fox	6	6	56	5.7	fox	5	29
5	domestic dog	18	6	30	3.8	dog	10	38
6	domestic dog	21	10	109	6.5	dog/Ariana	10	39
7	domestic dog	40	10	180	6.5	dog	11	41
8	domestic dog	12	6	28	7.4	dog	11	51
9	domestic dog	30	12	35	7.4	dog	11	48
10	domestic dog	12	7	35	3.7	unknown	13	52
11	mongoose	5	5	28	5	skunk	11	46
12	raccoon	5	5	30	4.9	raccoon	11	44
13	raccoon	7	7	28	4.9	raccoon	11	45
14	raccoon	14	5	56	4.9	raccoon	11	47
15	raccoon	61	19	442	6.7	raccoon	4	49
16	raccoon	30	10	350	5.9	raccoon	4	24
17	raccoon	10	5	60	*	raccoon	11	49
18	raccoon	6	13	28/65	5.5	dog	12	53
5	raccoon dog	6	9	124	2.8	coyote	10	38
19	red fox	36	6	34	4.7	fox	1	19
20	red fox	84	31	30	4	fox	2	20
21	red fox	66	25	730	4	fox	2	21
22	red fox	27	4	28	3.8	fox	1	22
23	red fox	13	4	28/180/360	3.2	fox	3	23
24	red fox	25	9	547	4.9	fox	4	26
25	red fox	18	6	2490	6.7	fox	4	31
26	red fox	25	0	28	5.7	fox	6	32
27	red fox	27	14	190	5.3	canine/coyote	7	33
28	red fox	8	2	45	5.1	canine/coyote	9	37
16	red fox	6	14	97	3	fox	10	38
29	red fox	16	8	107	3.3	fox	8	40
30	red fox	16	8	16	3.2	fox	8	35
28	striped skunk	3	2	45	5.1	canine/coyote	9	37
31	striped skunk	54	10	247	4.9	arctic fox	4	25
29	striped skunk	32	8	90/107	7.8	skunk	8	40
32	striped skunk	6	9	16	5.5	skunk	11	42
33	striped skunk	24	6	116	6.3	skunk	11	43
12	striped skunk	5	5	30	5	skunk	11	44
13	striped skunk	17	6	35	4.2	skunk	11	45
34	striped skunk	26	8	90	6.3	skunk	8	36
35	striped skunk	32	8	90	5.3	skunk	8	34

* Challenge dose given as ≥5 LD_50_. Facility Key: 1—Ministere de l’Agriculture, Centre National d’Etudes sur la Rage et la Pathologie des Animaux Sauvages, Malzeville, France; 2—Connaught Laboratories Limited, Ontario, Canada; 3—Department of Virology, Faculty of Veterinary Medicine, University of Liege, Brussels, Belgium; 4—Ontario Ministry of Natural Resources, Wildlife Research and Development Section, Trent University, DNA Building, Peterborough, Ontario, Canada; 5—Institute of Arctic Biology, University of Alaska, Fairbanks, AK, USA; 6—Laboratoire de Genetique, Dedex, France; 7—Institute of Molecular Virology and Cell Biology, Friedrich-Loeffler-Institute-Federal Research Institute for Animals Health, Germany; 8—Agriculture Canada, Animal Diseases Research Institute, NEPEAN, Ontario, Canada; 9—IDT Biologika GmbH, Am Pharmapark, 06855 Dessau-Rosslau, Germany; 10—AFSSA, National Research Laboratory on Rabies and Wildlife Diseases, WHO Collaborating Centre for Research and Management in Zoonoses Control, OIE Reference Laboratory on Rabies, EU Reference Institute for Rabies Serology, Malzeville, France; 11—Centers for Disease Control and Prevention, Atlanta, GA, USA; 12—Wistar Institute of Anatomy and Biology; 13—Department of Pathology, University of Georgia, Athens, GA, USA.

**Table 2:** Animal vaccine efficacy and challenge studies referenced for serology data as part of the empirical data analysis.

Study	Animal Species	Vacc.	Ctls.	Challenge after … Days	Challenge Dose (MICLD50 log)	Challenge Virus Strain	Facil.	Approval	Date (dd/mm/yy)
1	domestic dog	16	4	56	3.6	dog	IDT	42502-3-710 IDT	2/26/2014
2	mongoose	15	4	56	4.2	dog	IDT	42502-3-693 IDT	24/06/2013
3	mongoose	11	3	42	4	dog	IDT	42502-3-693 IDT	6/24/2013
4	raccoon	5	3	49	4.7	coyote	IDT	42502-3-582 IDT	28/10/2009
5	raccoon	3	-	56	5	coyote	IDT	42502-3-582 IDT	28/10/2009
6	raccoon	20	4	56	4.6	dog	IDT	42502-3-669 IDT	11/23/2012
7	raccoon	-	12	-	4.2–6.2	raccoon	IDT	42502-3-726 IDT	29/07/2014
8	raccoon	11	6	180	6.2	dog(fox)	NWRC	QA2278A	4/21/2014
9	raccoon	23	6	180	5.9	dog(fox)	NWRC	QA2278B	4/21/2014
10	raccoon dog	6	2	56	5	coyote	IDT	42502-3-669 IDT	11/23/2012
11	raccoon dog	30	12	190	0.7	fox	FLI	7221.3-2-007/14	4/1/2014
12	raccoon dog	-	22	-	2.3–3.0	fox	IDT	42502-3-741 IDT	8/17/2015
13	raccoon dog	16	2	28	2.0–2.7	fox	IDT	42502-3-761 IDT	17/08/2015
14	raccoon dog	-	20	-	1.7–2.7	fox	IDT	42502-3-761 IDT	17/08/2015
15	raccoon dog	30	12	183/184	3	fox	FLI	7221.3-2-018/15	17/06/2015
16	red fox	12	3	98	5	coyote	IDT	Unknown *	Unknown *
17	red fox	6	2	62	5	coyote	IDT	42502-3-582 IDT	28/10/2009
18	red fox	6	2	56	5	coyote	IDT	42502-3-669 IDT	11/23/2012
19	red fox	6	4	56	6.7	dog	IDT	42502-3-669 IDT	11/23/2012
20	red fox	9	2	56	4.6	fox	IDT	42502-3-669 IDT	11/23/2012
21	red fox	-	18	-	2.0–4.7	fox	IDT	42502-3-735 IDT	17/08/2015
22	red fox	20	4	50	3	fox	FLI	7221.3-2-005/16	1/28/2016
23	red fox	30	12	190	0.7	fox	FLI	7221.3-2-007/14	4/1/2014
24	striped skunk	8	2	42	5	coyote	IDT	42502-3-582 IDT	28/10/2009
25	striped skunk	5	3	58	4.7	coyote	IDT	42502-3-582 IDT	28/10/2009
26	striped skunk	6	3	56	5	coyote	IDT	42502-3-582 IDT	28/10/2009
27	striped skunk	16	4	28/56	4.6	dog	IDT	42502-3-669 IDT	11/23/2012
28	striped skunk	20	4	335	5.9	dog(fox)	NWRC	QA2278	8/15/2014

* At that time, a different procedure was followed (2007). Facility key: IDT—IDT Biologika GmbH, Germany; FLI—Friedrich Loeffler Institute, Germany; NWRC—National Wildlife Research Centre, Fort Collins, USA.

**Table 3:** Test characteristics and test agreement between the respective tests and the outcome of infection (survival vs. death), with two different cutoff values for the blocking ELISA (BioPro) and for RFFIT. For all results (OR = odds ratio, Se = sensitivity, Sp = specificity, PPV = positive predictive value, NPV = negative predictive value), the 95% confidence interval (CI) is provided.

Diagnostic Test	OR	(95% CI)	Se	(95% CI)	Sp	(95% CI)	PPV	(95% CI)	NPV	(95% CI)	*p* Value	Kappa	(95% CI)	Agreement
**blocking ELISA (70% Inhib)**	**18.54**	**4.982–78.59**	**0.4873**	**0.4486–0.5263**	**0.9512**	**0.8386–0.9913**	**0.9935**	**0.9768–0.9989**	**0.1074**	**0.07959–0.1435**	**<0.0001**	**0.016**	**0.062–0.126**	**Poor**
**blocking ELISA (40% Inhib)**	**56.39**	**21.5–148**	**0.9063**	**0.8567–0.9399**	**0.8537**	**0.7156–0.9312**	**0.9667**	**0.9292–0.9846**	**0.6604**	**0.5259–0.7731**	**<0.0001**	**0.681**	**0.564–0.799**	**Good**
Fox	126	14.48–1383	0.9825	0.9071–0.9991	0.6923	0.4237–0.8732	0.9333	0.8407–0.9738	0.9	0.5958–0.9949	<0.0001	0.741	0.527–0.955	good
Raccoon dog	∞	12.29–∞	0.8795	0.7922–0.9332	1	0.6756–1	1	0.95–1	0.4444	0.2456–0.6628	<0.0001	0.562	0.332–0.792	moderate
Mongoose	∞	9.259–∞	1	0.7225–1	1	0.6457–1	1	0.7225–1	1	0.6457–1	<0.0001	1.000	1.000-1.000	perfect
Skunk	6	0.8095–41.37	0.8	0.5481–0.9295	0.6	0.2307–0.9289	0.8571	0.6006–0.9746	0.5	0.1876–0.8124	0.1313	0.375	0.071–0.821	fair
Dog	N/A	N/A	0.375	0.1848–0.6136	N/A	N/A	1	0.6097–1	0	0–0.2775	>0.9999	0.000	0.000-0.000	poor
Raccoon	∞	2.432–∞	0.6364	0.3538–0.8483	1	0.6756–1	1	0.6457–1	0.6667	0.3906–0.8619	0.0128	-0.390	-0.676–-0.104	worse
**RFFIT (0.5 IU/mL)**	**32.1**	**10.2–101.1**	**0.7171**	**0.6519–0.7743**	**0.9268**	**0.8057–0.9748**	**0.98**	**0.9429–0.9945**	**0.3958**	**0.3038–0.4958**	**<0.0001**	**0.419**	**0.312–0.526**	**Moderate**
Fox	16.25	3.735–60.62	0.8298	0.6986–0.9111	0.7692	0.4974–0.9182	0.9286	0.8099–0.9754	0.5556	0.3372–0.7544	0.0001	0.526	0.285–0.767	moderate
Raccoon dog	∞	7.174–∞	0.7952	0.6962–0.868	1	0.6756–1	1	0.945–1	0.32	0.1721–0.5159	<0.0001	0.406	0.201–0.611	moderate
Mongoose	∞	1.121–∞	0.3793	0.2269–0.56	1	0.6457–1	1	0.7412–1	0.28	0.1428–0.4758	0.0756	0.192	0.037–0.348	poor
Skunk	∞	4.573–∞	0.8571	0.6536–0.9502	1	0.5655–1	1	0.8241–1	0.625	0.3057–0.8632	0.0009	0.698	0.391–1.000	good
Dog	N/A	N/A	0.5	0.28–0.72	N/A	N/A	1	0.6756–1	0	0–0.3244	>0.9999	0.000	N/A	poor
Raccoon	∞	0.6622–∞	0.2941	0.1328–0.5313	1	0.6756–1	1	0.5655–1	0.4	0.2188–0.6134	0.1399	0.211	0.013–0.408	fair
**RFFIT (0.25 IU/mL)**	**24.83**	**9.924–59.72**	**0.8098**	**0.7505–0.8576**	**0.8537**	**0.7156–0.9312**	**0.9651**	**0.926–0.9839**	**0.473**	**0.3634–0.5852**	**<0.0001**	**0.502**	**0.382–0.622**	**Moderate**
Fox	5.415	1.584–18.03	0.7719	0.6479–0.8616	0.6154	0.3552–0.8229	0.898	0.7824–0.9556	0.381	0.2075–0.5912	0.0152	0.313	0.072–0.554	fair
Raccoon dog	∞	9.542–∞	0.8434	0.7502–0.9061	1	0.6756–1	1	0.948–1	0.381	0.2075–0.5912	<0.0001	0.486	0.265–0.708	moderate
Mongoose	∞	2–∞	1	0.7412–1	0.8571	0.4869–0.9927	0.9167	0.6461–0.9957	1	0.6097–1	0.0004	0.880	0.653–1.000	very good
Skunk	∞	5.995–∞	0.9048	0.7109–0.9831	1	0.5655–1	1	0.8318–1	0.7143	0.3589–0.9492	0.0003	0.785	0.506–1.000	good
Dog	N/A	N/A	0.875	0.6398–0.9778	N/A	N/A	1	0.7847–1	0	0–0.8223	>0.9999	0.000	N/A	poor
Raccoon	∞	1.618–∞	0.4706	0.2617–0.6904	1	0.6756–1	1	0.6756–1	0.4706	0.2617–0.6904	0.0261	0.363	0.106–0.619	fair

## Supplementary Materials

The following are available online at http://www.mdpi.com/2414-6366/2/3/31/s1.
